# Estimating the Impact of Statewide Policies to Reduce Spread of Severe Acute Respiratory Syndrome Coronavirus 2 in Real Time, Colorado, USA

**DOI:** 10.3201/eid2709.204167

**Published:** 2021-09

**Authors:** Andrea G. Buchwald, Jude Bayham, Jimi Adams, David Bortz, Kathryn Colborn, Olivia Zarella, Meghan Buran, Jonathan Samet, Debashis Ghosh, Rachel Herlihy, Elizabeth J. Carlton

**Affiliations:** Colorado School of Public Health, Aurora, Colorado, USA (A.G. Buchwald, O. Zarella, M. Buran, J. Samet, D. Ghosh, E.J. Carlton);; Colorado State University, Fort Collins, Colorado, USA (J. Bayham);; University of Colorado, Denver, Colorado, USA (J. Adams);; University of Colorado, Boulder, Colorado, USA (D. Bortz);; University of Colorado School of Medicine, Aurora (K. Colborn);; Colorado Department of Public Health and Environment, Denver (R. Herlihy)

**Keywords:** severe acute respiratory syndrome coronavirus 2, SARS-CoV-2, coronaviruses, viruses, coronavirus disease, COVID-19, infectious disease transmission, social distancing, mobility, mathematical models, real time, respiratory infections, zoonoses, Colorado, United States

## Abstract

The severe acute respiratory syndrome coronavirus 2 (SARS-CoV-2) pandemic necessitated rapid local public health response, but studies examining the impact of social distancing policies on SARS-CoV-2 transmission have struggled to capture regional-level dynamics. We developed a susceptible-exposed-infected-recovered transmission model, parameterized to Colorado, USA‒specific data, to estimate the impact of coronavirus disease‒related policy measures on mobility and SARS-CoV-2 transmission in real time. During March‒June 2020, we estimated unknown parameter values and generated scenario-based projections of future clinical care needs. Early coronavirus disease policy measures, including a stay-at-home order, were accompanied by substantial decreases in mobility and reduced the effective reproductive number well below 1. When some restrictions were eased in late April, mobility increased to near baseline levels, but transmission remained low (effective reproductive number <1) through early June. Over time, our model parameters were adjusted to more closely reflect reality in Colorado, leading to modest changes in estimates of intervention effects and more conservative long-term projections.

Mathematical transmission models are useful tools for predicting the magnitude, duration, and severity of the severe acute respiratory coronavirus 2 (SARS-CoV-2) pandemic. However, widely used national-level models might not capture regional heterogeneity. The coronavirus disease (COVID-19) outbreak in Colorado, USA, has been the subject of numerous discrepant projections from the Institute for Health Metrics and Evaluation and other modeling groups (*1*), which might have structural and data source explanations, highlighting the need for ensuring that models are fit to local epidemiologic data (*2**–**4*).

We report on our experience using a locally tailored model to inform policy in Colorado. Social distancing policies, intended to decrease contact rates, have been cornerstone public health tools for pandemic control, and these strategies have been adopted to control SARS-CoV-2 globally (*2**,**5*). Until recently, evidence of the effects of social distancing has come primarily from studies of the consequences of school and transit closures on influenza transmission (*3**,**4**,**6*). Early evidence suggests that social distancing policies can suppress transmission of SARS-CoV-2 (*7**,**8*), and recent evidence suggests a strong correlation between mobility and transmission reduction (*9*). However, these studies largely focused on periods when social distancing policies were in place, leaving critical questions unanswered regarding how long populations will comply with such measures and what happens when policies are relaxed.

One of the defining characteristics of the COVID-19 pandemic is the need for rapid response in the face of imperfect and incomplete information. Mathematical models of infectious disease transmission can be used in real-time to estimate parameters, such as the effective reproductive number (R_e_) and the efficacy of current and future intervention measures, providing time-sensitive data to policy-makers (*10*). We describe development of such a model, in close collaboration with the Colorado Department of Health and Environment and the Governor’s office, to gauge the current and future effects of early policies to decrease social contacts and, later, the gradual relaxing of stay-at-home orders.

We developed a compartmental susceptible-exposed-infected-recovered (SEIR) model calibrated to statewide COVID-19 case and hospitalization data to estimate changes in the contact rate and the R_e_ after emergence of SARS-CoV-2 and the implementation of statewide social distancing policies in Colorado. We supplemented model estimates with an analysis of mobility by using mobile-device location data. Estimates were generated in near real time, at multiple time-points, with a rapidly evolving understanding of SARS-CoV-2. At each time point, we generated projections of the possible course of the outbreak under future social distancing scenarios. Findings were regularly provided to key Colorado decision-makers. We present estimates generated at multiple time points to document how our model, estimates and projections evolved over time. Although our analysis is specific to Colorado, our experience highlights the need for locally calibrated transmission models to inform public health preparedness and policymaking, along with ongoing analyses of the impact of policies to slow the spread of SARS-CoV-2.

## Methods

### COVID-19 Timeline and Policies

The first SARS-CoV-2 case was reported in Colorado on March 5, 2020, and a rapid succession of policies to control transmission followed (Table 1). The Colorado governor formally declared a disaster emergency on March 11. During March 14‒April 16, a total of 35 executive orders were mandated to curb SARS-CoV-2 transmission, including school closures, reduction in workforce percentages, and shelter-in-place (stay-at-home) orders. In conjunction with state executive orders, the Colorado Department of Health and Environment issued orders closing restaurants, bars, and other congregate environments on March 17 and prohibiting gatherings of >10 persons on March 19. A state-wide stay-at-home order was in effect during March 26‒April 26. Transition to a less restrictive phase, safer-at-home, began on April 27, which enabled some businesses to reopen with restrictions. The metropolitan Denver counties, comprising ≈50% of the population of Colorado, were under extended stay-at-home orders until May 8.

**Table 1:** Key state-level events, executive orders, and policies directed at controlling transmission of SARS-CoV-2, Colorado, USA, 2020*

Policy/event	Description	Date announced	Policy effective date	Policy effective until
First case of COVID-19	First case of infection with SARS-CoV-2 reported	Mar 5	NA	NA
Executive Order D 2020 003	Disaster emergency	Mar 10	Mar 11	Apr 11
Executive Order D 2020 004	Ski resort closure	Mar 14	Mar 15	Mar 22
Executive Order D 2020 006	Extension of ski resort closure	Mar 18	Mar 18	Apr 17
CDPHE Order 20–22	Closure of bars, restaurants, theaters, gymnasiums, and casinos	Mar 16	Mar 17	Apr 16
CDPHE Order 20–23	Prohibition of >10 person gatherings	Mar 19	Mar 19	Apr 19
Executive Order D 2020 007	School closures during Mar 18‒Apr 17	Mar 18	Mar 18	Apr 17
Executive Order D 2020 013	Reduction of in-person workforce by 50%	Mar 22	Mar 24	May10
Executive Order D 2020 017	Stay-at-home order: directive to require all residents of Colorado to stay home unless in pursuant of essential items (i.e., food) or working for critical businesses and ordering noncritical businesses to close temporarily.	Mar 25	Mar 26	Apr 11
Executive Order D 2020 021	Extension to school closures until Apr 30	Apr 1	Apr 1	May 1
Executive Order D 2020 024	Stay-at-Home extension	Apr 6	Apr 6	Apr 26
Executive Order D 2020 039	Ordering workers in critical businesses and government functions to wear nonmedical face coverings	Apr 17	Apr 17	May 17
Executive Order D 2020 041	Suspension of school closures until end of school year	Apr 22	Apr 22	May 20
Executive Order D 2020 044	Safer at home: All susceptible persons and those who have COVID-19 instructed to stay at home. State residents directed to limit interactions, only travel for essential needs, and limit gatherings to <10 people in public and private spaces. Nonmedical mask coverings recommended. Retail businesses can open for curbside delivery, elective medical, dental, and veterinary surgeries and procedures resume. Retail businesses and personal services (e.g., salons) can open. Offices can open at 50% capacity.†	Apr 26	Apr 27–May 4	May 27
Executive Order D 2020 058	Disaster emergency extension	May 7	May 7	Jun 6
Executive Order D 2020 067	Extending EO D 2020 039, ordering workers in critical businesses and government, to wear nonmedical face coverings	May 16	May 16	Jun 16
Executive Order D 2020 079	Extension to EO D 2020 044: Safer at Home to permit public places to offer outdoor dining, and limited indoor dining	May 25	May 25	Jun 1

*COVID-19‒relevant executive orders are detailed in (*11*) and CDPHE policies are described in (*12*). CDPHE, Colorado Department of Public Health and Environment; COVID-19, coronavirus disease; EO, executive order; NA, not applicable; SARS-CoV-2, severe acute respiratory syndrome coronavirus 2. †Retail business and personal services were permitted to open on May 1, offices were permitted to open at 50% capacity on May 4. All other measures went into effect April 27.

### Reported Case and Hospitalization Data

Hospitalization data are a robust indicator of transmission trends compared with reported case data because reported case data are sensitive to testing capacity. However, because COVID-19 hospitalization data were sparse early in the epidemic, we initially fit models to reported COVID-19 cases from the Colorado Electronic Disease Reporting System (CEDRS). We fit models to the daily number of symptom onsets to reflect a biologically meaningful process (report date can be sensitive to testing lags). Missing onset dates were imputed as report date minus 7 days, the median onset-to-report lag. In May, we began fitting models to the daily number of hospitalized COVID-19 patients because we suspected that reported cases captured a variable proportion of infections over time because of increases in testing capacity. Daily hospital census records were obtained from EMResource (https://emresource.juvare.com). Because EMResource appeared to underreport COVID-19 hospitalizations during March, we inferred COVID-19 hospitalizations by using CEDRS before April 8.

### Model Description

We used a deterministic age-structured SEIR model with 3 age groups (<30, 30–59, and >60 years of age) to estimate key model parameters and project the number of COVID-19 hospitalizations. In the model, we assume a single virus introduction event occurring on January 24, a date extrapolated from the first reported cases in Colorado.

In the model, the probability that an infected person shows development of symptoms (*13*) and needs hospitalization or ICU care is age dependent (*14*). All persons have an equal probability of exposure and infection, regardless of age. Initially, we used published estimates (*15*) for the proportion of symptomatic case-patients requiring hospitalization and critical care. Starting in May, with sufficient hospitalization data, we generated Colorado-specific estimates of the probability of hospitalization and critical care among case-patients by using model-fitting approaches, which enabled us to better account for underlying health status and patterns of care in Colorado (*16*).

**Figure 1:**
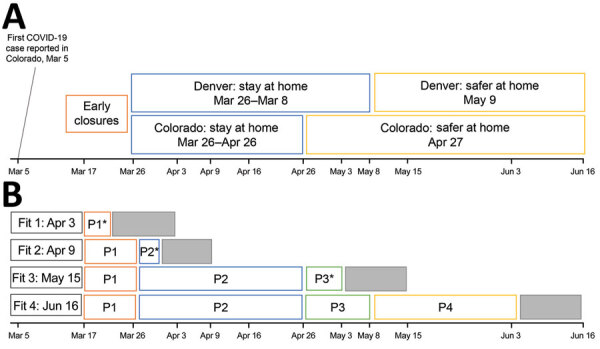
Emergence of COVID-19, Colorado, USA, 2020, showing policy-based responses (A) and definition of 4 distinct social distancing phases (B) corresponding with early closures (phase 1, March 17‒25); statewide stay-at-home (phase 2, March 26‒April 26), statewide partial transition to safer-at-home (phase 3, April 27–May 8); statewide safer-at-home (phase 4, May 5–June 3). Social distancing parameters were estimated at 4 points during March‒June by using model fitting procedures and reported case data (fits 1 and 2) and hospital census data (fits 3 and 4). In light of the 5.1 day mean incubation period, the ≈7-day lag between symptom onset and case report, and the ≈8-days between symptom onset and hospitalization based on state records, there are ≈12 and 13 day lags between infection and case report, and infection and hospitalization, respectively (gray boxes). Thus, at each model fit, we could estimate social distancing parameters reflecting transmission conditions up to 12 (fits 1 and 2) or 13 (fits 3 and 4) days before the fit date. Asterisks (*) indicate estimate generated for only part of the period. COVID-19, coronavirus disease; P, phase.

The model includes 3 types of transmission-reducing parameters: social distancing, mask wearing, and self-isolation of symptomatic persons. Social distancing was modeled as a reduction in the contact rate between susceptible and infectious persons by multiplying the transmission parameter, β, by (1 ‒ social distancing). We defined contacts as interactions that could enable spread of infections from an infected person to a susceptible person. The term social distancing is used to encompass all contact-reducing behaviors and policies, including working from home, school closures, maintaining physical distancing, socializing outdoors (vs. indoors), and increased hygiene. Social distancing was modeled in phases coinciding with major events and policy measures (Figure 1). Phase 1 (March 17–25) corresponds with mid-March policies and increasing public concern regarding COVID-19, phase 2 (March 26–April 26) corresponds with the state-wide stay-at-home order, phase 3 (April 27–May 8) is the period when half the state transitioned to safer-at-home, and phase 4 (May 9–June 3) is the period when safer-at-home was in effect statewide.

We added mask wearing to the model in May (fits 3 and 4) in response to increasing evidence that masks are effective for controlling transmission (*17*,*18*). We modeled the effect of mask wearing as a reduction in the spread of infections from asymptomatic and presymptomatic persons to nonhousehold contacts. More recent evidence suggests that masks might also protect the wearer, an added benefit not considered here (*19*). The effectiveness of mask wearing depends on the ability of the mask to trap infectious particles and the proportion of the population wearing masks. We assume in the model that masks made from household materials are ≈50% effective in trapping infectious particles when worn properly (*17*,*18*). Previous studies estimated that ≈23% of contacts occur at home (*20*). Because persons spent more time at home during the pandemic, we assumed that 67% of the contact of an individual is with nonhousehold members. We assumed that mask wearing was uniform across asymptomatic and presymptomatic persons and fit the proportion of the population wearing masks beginning on April 4, the date of the governor’s press conference advising persons living in Colorado to wear masks. Because some transmission might also occur by fomites, we modeled mask effectiveness as a net 27% reduction in infectiousness among asymptomatic persons wearing masks. In addition, we assume mask wearing decreases transmission by presymptomatic persons (*21*,*22*); this is modeled as a 3.4% reduction in infectiousness for symptomatic persons wearing masks (assuming that symptomatic persons are asymptomatic on 1 of 8 infectious days). This model does not account for potential reduction in infectiousness by symptomatic persons who are assumed to isolate (*23*).

We modeled self-isolation assuming that a proportion of symptomatic case-patients self-isolate 24 hours after the onset of symptoms, and that self-isolation reduces transmission by symptomatic persons to nonhousehold contacts. This assumption is modeled as a 59% reduction in contacts by symptomatic persons who self-isolate. Self-isolation begins in the model on March 5 and the proportion of symptomatic persons who self-isolate is fit to the data.

### Estimating Social Distancing and Other Transmission-Reducing Interventions

We inferred the effect of social distancing and other interventions on transmission by using an algorithm-based optimization procedure at 4 different time points from April through June. We used the same approaches to estimate parameters that might vary regionally or for which there was considerable uncertainty in the literature (Table 2). We identified best-fitting parameter values by using a least-squares cost function that minimized difference between the model-estimated and observed number of reported SARS-CoV-2 cases in Colorado (fits 1 and 2) and the observed number of COVID-19 hospitalizations (fits 3 and 4). When fitting to cases (fits 1 and 2), it was necessary to also fit a parameter for the estimated probability that cases would be detected by state surveillance. We minimized the cost function by using a 2-stage fitting algorithm in R (*27*) and used the FME package (*28*) by first applying a pseudo-random optimization algorithm (*29*) to find a region of minimum difference between the model and the data. The second phase used least-squares optimization applying the Levenberg-Marquardt algorithm (*30*). We calculated 95% CIs by using a Markov Chain Monte Carlo simulation with an adaptive Metropolis algorithm with 1,500 iterations (*28*). This method obtains 95% CIs by sampling from a Gaussian distribution around the mean trajectory of the ordinary differential equation model. By the end of March, the differential sensitivity matrix was full rank, and thus all 6 parameters were identifiable for all datasets used (Appendix Figure 3).

**Table 2:** Model-estimated levels of social distancing, mask wearing and other parameter values at 4 time points over the course of the SARS-CoV-2 epidemic, Colorado, USA, 2020*

Characteristic	Range of possible values and sources (ref.)	Fitted value as of Apr 3† (95% CI)	Fitted value as of Apr 16† (95% CI)	Fitted value as of May 15† (95% CI)	Fitted value as of Jun 16† (95% CI)
Estimated effectiveness of social distancing					
Phase 1: early closures, Mar 17‒25, %‡	10‒70	45 (42‒53)	65 (63‒72)	52 (49‒66)	52 (52‒53)
Phase 2: state-wide stay-at-home, Mar 26–Apr 26, %	50‒99	NA	76 (72‒77)	80 (80‒83)	81 (80‒82)
Phase 3: half of state under stay-at-home, half transitioned to safer at home, Apr 27–May 8, %	45‒99	NA	NA	80 (78‒84)	85 (83‒90)
Phase 4: statewide safer at home, May 9–Jun 3, %§	NA	NA	NA	NA	90 (85‒93)
Proportion of population wearing masks starting Apr 4	0.1‒0.8	NA	NA	0.4 (0.11‒0.64)	0.40 (0.15‒0.76)
Other parameter values					
Rate of infection	0.2‒0.6 (*24*)	0.41 (0.39‒0.42)	0.50 (0.49‒0.51)	0.48 (0.46‒0.49)	0.48 (0.48‒0.48)
Reduction in infectious contacts due to symptomatic persons who self-isolate after March 5	0.3‒0.8 (*15*)	0.38 (0.22‒0.43)	0.47 (0.34‒0.50)	0.30 (0.29‒0.31)	0.32 (0.32‒0.32)
Ratio of infectiousness for symptomatic vs. asymptomatic persons	1.0‒4.0 (*25*,*26*)	2.27 (2.22‒2.29)	1.50 (1.35‒1.56)	1.65 (1.60‒1.77)	1.68 (1.67‒1.69)
Probability that symptomatic cases are identified by state surveillance	0.05‒0.6 (*24*)	0.28 (0.16‒0.45)	0.33 (0.27‒0.44)	NA	NA

*COVID-19, coronavirus disease; NA, not available; ref, reference; SARS-CoV-2, severe acute respiratory syndrome coronavirus 2. †Fit 1 uses all reported SARS-CoV-2 cases as of Apr 3 with an onset date of Mar 26 or earlier; fit 2 uses all reported cases as of Apr 16 with an onset date of Apr 8 or earlier; fit 3 uses COVID-19 hospital census through May 15; fit 4 uses hospital census data through Jun 16. ‡For the purpose of model fitting, phase 1 social distancing was modeled by using a start date of Mar 17, which corresponded with the closure of bars, restaurants, casinos, and many public schools in the state. §The statewide Safer at Home policy remained in effect through the summer, but because of the ≈5.1-d mean incubation period and the 8-d lag between symptom onset and hospitalization, we were able to generate transmission estimates through Jun 3.

Because of the median 7-day lag between symptom onset and reporting, on April 3 (fit 1), we included cases that had a symptom onset date through March 26 for model fitting, which enabled us to generate a preliminary estimate of phase 1 social distancing. On April 16 (fit 2), we included cases that had an onset date through April 9 for model fitting, which enabled us to refine estimates of social distancing during phase 1 and generate preliminary estimates of social distancing during phase 2. We generated a preliminary estimate of phase 3 social distancing on May 15 (fit 3) and then re-estimated on June 16 (fit 4), when social distancing during phase 4 was estimated. We estimated the proportion of the population wearing masks in fits 3 and 4 with the effectiveness assumptions defined and estimated the effective reproduction number (R_e_) from the model output. We additionally estimated the overall number of hospitalizations prevented as a result of decreasing contacts by comparing the fitted model on June 16 with a reference scenario assuming no reduction of the contact rate (social distancing = 0%).

### Projections of COVID-19 Hospitalizations and ICU Need

We used the fitted parameters to generate projections of future hospital and critical care needs under different scenarios. Changes in social distancing were implemented beginning 2 weeks after the date of model fitting to account for lags in policy implementation. All other parameters were held fixed as estimated from the model. Preparing for and meeting ICU load was a critical decision-making issue.

### Population Mobility

We used SafeGraph (https://www.safegraph.com) data to examine changes in mobility in Colorado from March through June. Specifically, we used an aggregated and anonymized measure of time away from home reported at the census block group. We calculated changes in mobility as a percent decrease in time away from home relative to pre-epidemic baseline: January 29‒February 15 (mean 2.3 hours).

We examined the relationship between mobility and transmission by calibrating the transmission parameter, β, conditional on time away from home at baseline. We then projected the model forward, enabling the parameter for the daily time away from home to change according to observed mobility data. These projections assume no other transmission reducing behavior (i.e., no mask wearing or self-isolation) to avoid conflating parametric assumptions with changes in observed mobility, nor changes to any other aspects of the model.

## Results

### Estimating Efficacy of Social Distancing and Other Transmission-Reducing Interventions

**Figure 2:**
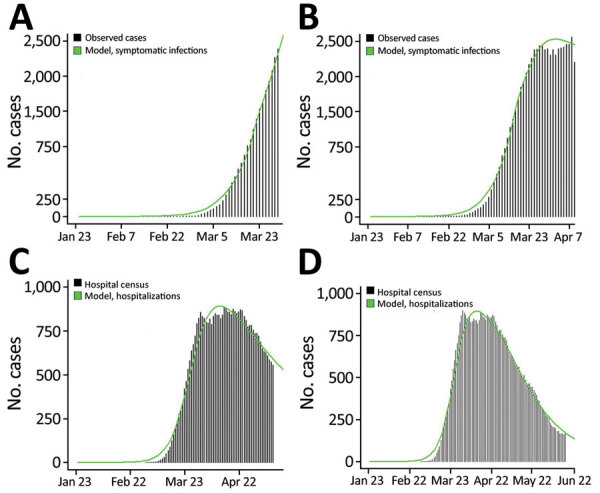
Observed (black bars) versus model-estimated (green line) number of reported coronavirus disease cases (panels A, B) and hospitalizations (panels C, D), Colorado, USA, 2020, based on models calibrated at 4 points in the early months of the epidemic. Model-based estimates were generated by using an age-structured susceptible-exposed-infected-recovered model, and best-fit parameter values were estimated based on observed data shown. Reported cases are shown by using symptom onset date or report date minus 7 days if onset date was missing, in accordance with onset to report lags for Colorado during this period.

**Figure 3:**
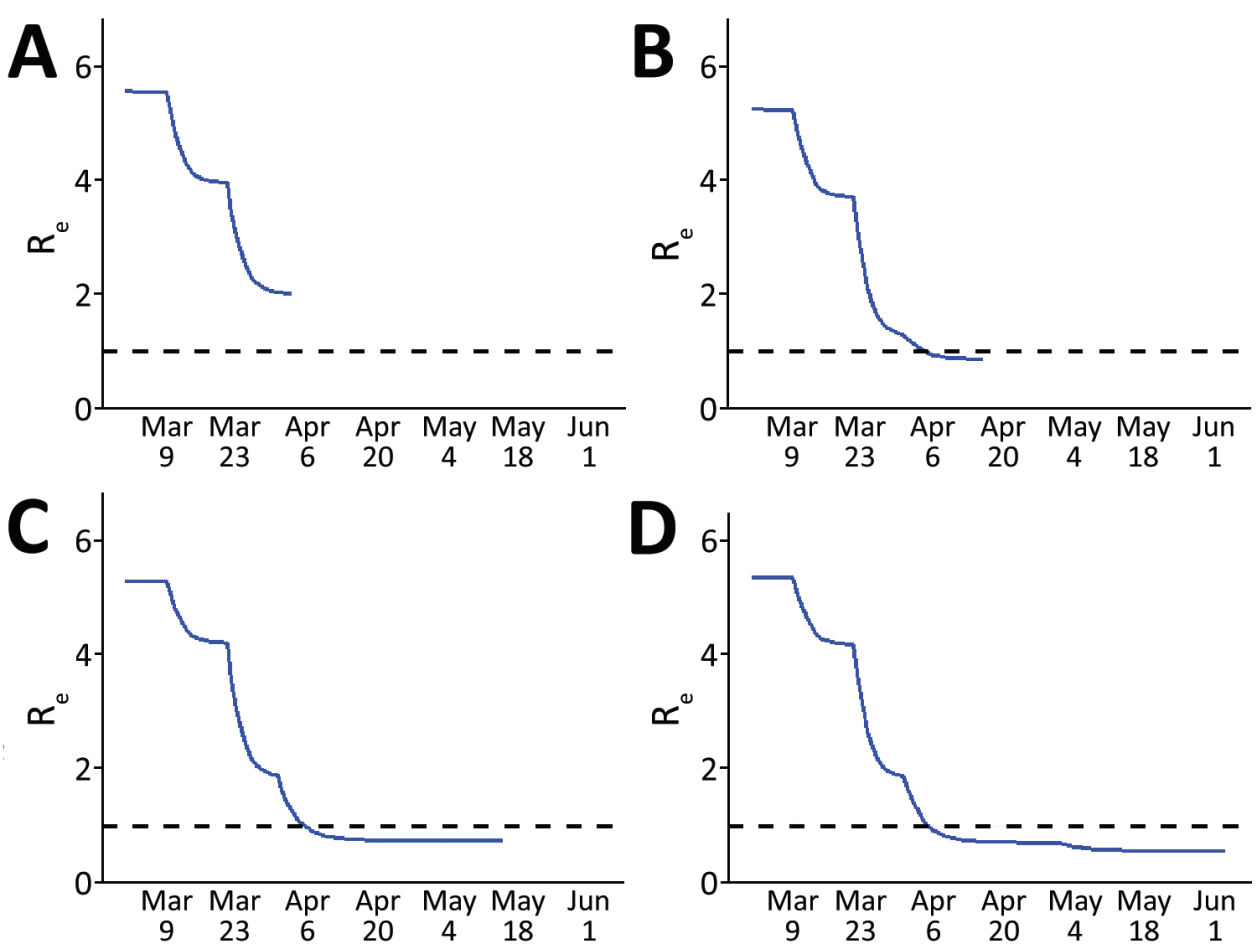
Estimated R_e_ over time, Colorado, USA 2020, based on susceptible-exposed-infected-recovered models fit to data at 4 time points in the early months of the epidemic. The reproductive number was estimated from model output at the time of each fit. A) Fit 1 on April 3; B) fit 2 on April 16; C) fit 3 on May 15; D) fit 4 on June 16. Dashed lines indicate an R_e_ of 1, below which the rate of new infections decreases and above which the rate of new infections increases. R_e_, effective reproductive number.

On April 3 (fit 1), we generated a preliminary estimate of social distancing during phase 1, which equated to a ≈45% decrease in the contact rate (Table 2; Figure 2), and R_e_ decreased from 5.3 to 2.4 (Figure 3). Because of the ≈12-day lag between infection and case report, this preliminary estimate was through March 21. On April 16 (fit 2), with more complete case data, the updated estimate of social distancing during phase 1 was greater: a 65% decrease in the contact rate. At this point, we generated a preliminary estimate of the level of social distancing during the first 2 weeks of phase 2 (March 26‒April 4): 76%. R_e_ was estimated to be 0.9. Incorporating increasing data and using COVID-19 hospitalizations instead of case reports, on May 15 (fit 3), we re-estimated phase 1 and phase 2 parameters, which indicated transmission reduction had been more moderate initially (social distancing = 52% for phase 1), and greater for phase 2 (social distancing = 80%). On May 15, which was 18 days after the end of the stay-at-home order, there was no evidence of an increase in hospitalizations or contact rate due to decreased restrictions, noting that because of the ≈13-day lag between infection and hospitalization, this estimate reflects transmission through May 2. A month later on June 16 (fit 4), a greater decrease in transmission was estimated for phase 3 (social distancing = 85%), and the estimated decrease in contact rates during safer-at-home (phase 4) was 90%. Estimated R_e_ decreased to 0.6 during phase 4.

### Estimated Reduction in Hospitalizations from Decreased Contacts

As of June 16, a total of 5,272 COVID-19 hospitalizations in Colorado had been reported to CEDRS, and EMResource data strongly suggested underreporting of COVID-19 hospitalizations to CEDRS during April and May. Using CEDRS and EMResource data, we found that the SEIR model estimated a cumulative of 5,344 COVID-19 hospitalizations in Colorado by Jun 16 (Figure 2, panel D). Assuming that no measures had been taken to alter individual behavior or risk perception (social distancing 0% throughout), we estimate that >173,000 hospitalizations would have occurred by that same date, suggesting that ≈97% of potential hospitalizations were avoided as a result of decreases in effective contacts.

### Projecting Hospitalizations and ICU Need

**Figure 4:**
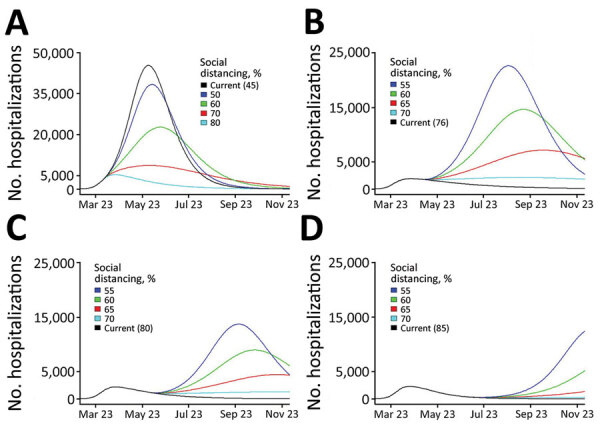
Projected coronavirus disease hospitalizations, Colorado, USA, 2020, if current trajectory continued (black line) and for a range of social distancing scenarios (colored lines) generated by models calibrated at 4 time points during April‒June (fit 1: Apr 3; fit 2: April 16; fit 3: May 15; fit 4: June 16). Current trajectory was based on estimated parameters generated for each fit. Social distancing is modeled as a percent reduction in the contact rate (from baseline), and changes in social distancing are introduced 2 weeks after model fitting date. All other fitted parameters are held at the estimated values for each fit. Because peak hospitalization estimates from fit 1 were substantially higher than estimated for later fits, the y-axis is scaled to 50,000 as opposed to 25,000 for fits 2–4. Numbers in parentheses are current values.

**Figure 5:**
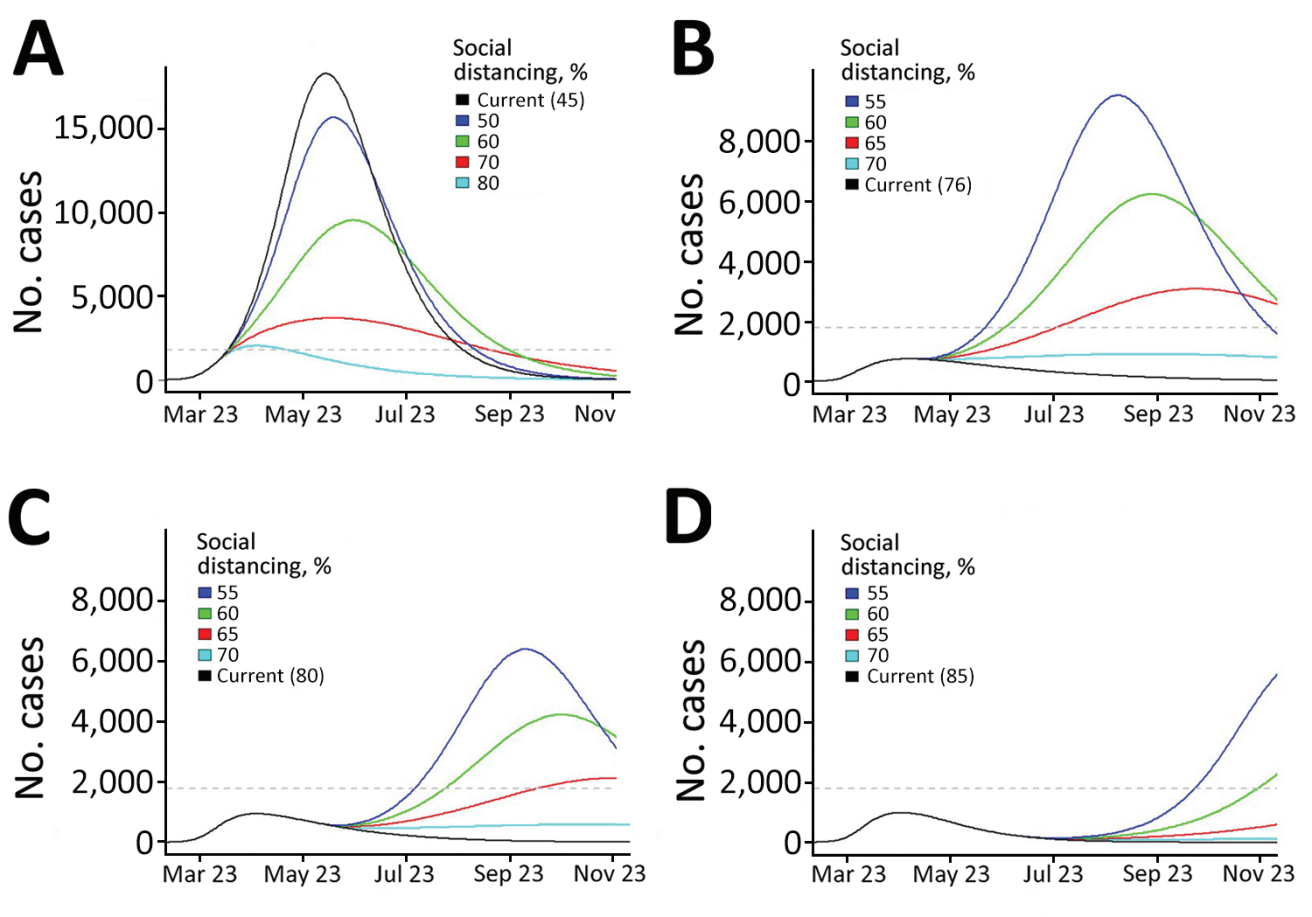
Projected coronavirus disease ICU needs, Colorado, USA, 2020, if current trajectory continues (black line) and for a range of social distancing scenarios (colored lines) generated by using models calibrated at 4 time points during April‒ June (fit 1: Apr 3; fit 2: April 16; fit 3: May 15; fit 4: June 16). Current trajectory was based on estimated parameters generated for each fit. Social distancing is modeled as a percent reduction in the contact rate from baseline, and changes in social distancing are introduced 2 weeks after model fitting date. All other fitted parameters are held at the estimated values for each fit. Because ICU estimates from fit 1 were substantially higher than for later fits, the y-axis is scaled to 20,000 as opposed to 10,000 for fits 2–4. Colorado estimated ICU capacity (1,800 beds) is indicated by the dashed gray horizontal line. ICU, intensive care unit. Numbers in parentheses are current values.

We provide projected hospitalizations (Figure 4) and ICU need (Figure 5) that were generated from each of the 4 model fits. Fit 1, produced with the least available data, substantially overestimated hospitalizations and ICU need compared with later fits and predicted that ICU capacity would be exceeded even if 80% contact reduction was achieved. As data accumulated and transmission slowed in the state, the estimated peaks under all possible levels of social distancing decreased and shifted later in the year, and projections indicated contact reduction would need to remain at or above ≈70% to prevent exceeding ICU capacity.

### Association between Changes in Mobility and Contact Rates

**Figure 6:**
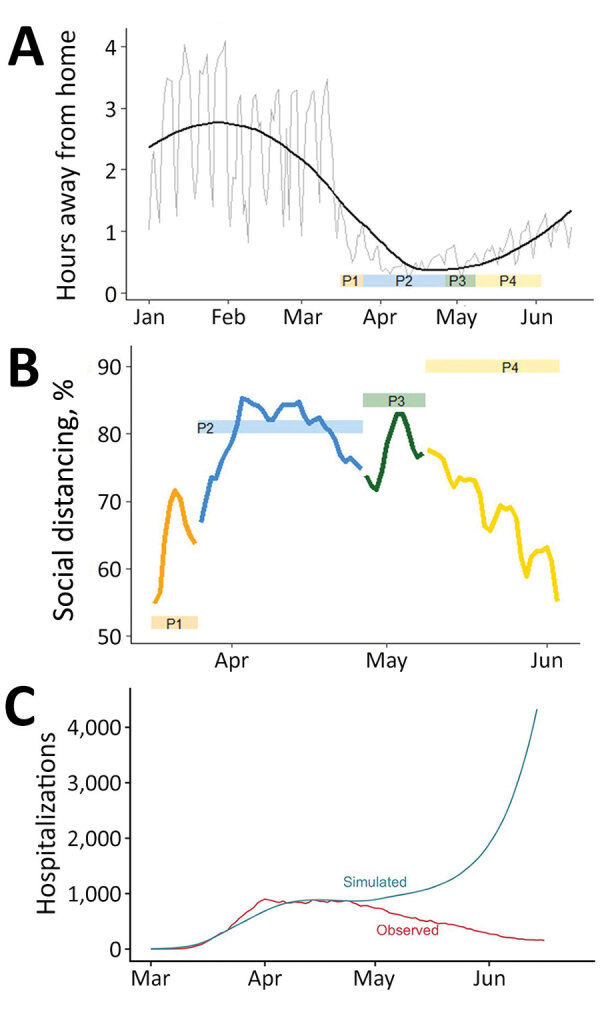
Changes in population mobility before and after emergence of coronavirus disease, Colorado, USA, 2020, and comparison between mobility and estimated social distancing. A) Changes in mobility measured by the number hours spent away from home per day (source: SafeGraph, https://www.safegraph.com). Gray line indicates daily observations, and black line indicates a smoothed line using locally estimated scatterplot smoothing in R (https://www.r-project.org). The ribbon at the bottom indicates the 4 social distancing phases. B) Comparison between susceptible-exposed-infected-recovered model-estimated social distancing (colored boxes) and reduction in mobility relative to the preintervention period, January 29–February 15 (colored lines). Colors correspond to the 4 social distancing phases. Reductions in mobility are calculated as percentage decreases in time away from home relative to pre-epidemic baseline. C) Observed hospitalization data (red) and the simulated hospitalizations based on time away from home relative to a baseline mean during January 29–February 15. In the simulation, it is assumed there is no self-isolation of symptomatic infectious, no mask wearing, and no other transmission reduction to highlight the role of the mobility data in the simulation. P, phase.

Residents of Colorado decreased activity outside the home starting in early March (Figure 6, panel A). The trends in mobility data suggest that, on average, the time spent away from home decreased by ≈60% from February to mid-April. Time away from home began to increase in late April, before the end of statewide stay-at-home orders, and increased steadily through June. Mobility metrics initially reinforced estimated social distancing levels, and percent reduction in time away from home coincided with estimated transmission reduction resulting from social distancing (Figure 6, panel B). However, increased time away from home in late April contrasted with estimated infectious contact reduction, which remained high through June. We compared hospitalizations simulated from mobility data to observed and observed a relatively strong association up until late April (Figure 6, panel C). After that, the modeled and observed trends decoupled, indicating that other behaviors or interventions not captured by mobility played a major role in transmission reduction.

## Discussion

We used an SEIR transmission model, calibrated to local COVID-19 case and hospital data, to estimate the collective impact of individual behaviors and public policy measures in reducing COVID-19 transmission in Colorado during 2020, providing time-sensitive estimates of the pandemic trajectory to policy-makers to assist in decision-making. COVID-19 policies introduced during March and April were followed by substantial decreases in contact rates and suppression of the R_e_ well below 1, in agreement with other studies of nonpharmaceutical interventions to decrease SARS-CoV2 transmission (*31**–**34*). These values remained low after the stay-at-home order was lifted and mobility increased.

The continued suppression of transmission might be explained, in part, by transmission control policies that remained in effect and/or were implemented after stay-at-home ended: outbreak prevention and control strategies in long-term care facilities persisted, capacity limits were implemented for businesses and restaurants, and mass gatherings were banned. Moreover, the state reopened slowly during April and May. Given the typical lags between infection and hospitalization, the estimates do not capture the impact of reopening measures implemented during or after late May (e.g., restaurant openings).

Mobility, assessed by using mobile-device data, generally reflected state-level policy during March‒June but mirrored transmission only in the early months of the pandemic. Mobility decreased rapidly in March in concert with early transmission control policies and the statewide stay-at-home order, and mobility increased as social distancing policies were relaxed. Consistent with our findings, others have found that US population mobility was reactive to policy during March: greater perceived disease prevalence and governmental stay-at-home orders resulted in less mobility and social interaction (*35**–**37*). However, in Colorado, lifting stay-at-home orders and concurrent increases in mobility do not appear to have led to increased transmission, indicating the limitations in using mobility data to predict transmission. These results warrant further investigation in other contexts to help clarify the utility of mobility data in SARS-CoV-2 forecasting, particularly during reopening phases.

Mobility data can be used to estimate when and where persons are congregating, a precondition for transmission, but do not sufficiently capture behaviors such as mask wearing, physical distancing, or moving activities outside. Those individual behaviors can play a critical role in spreading infections, and understanding what drives those behaviors can improve epidemic response. Public perception of and reaction to perceived risk is multifactorial, and, although clearly influenced by policy, in a time of heightened fear, local policy probably plays only a partial role in determining risk-reducing behavior. Rapid and frequent introduction of COVID-19‒related policy measures and public communication by government officials at the national, state, and local scales probably increased fear and public risk perception regarding COVID-19 transmission, and contributed to adoption of transmission-reducing behaviors before the start of and beyond the end of stay-at-home orders (*38*). Conversely, persons might perceive decreased risks and abandon risk-reducing behaviors when transmission control policies are relaxed, a phenomenon we suspect contributed to an increase in COVID-19 transmission in Colorado during July 2020 (*39*). Research on how risk perception and behavior fluctuates in relationship to the epidemic trajectory can improve communication and policy making.

Real-time estimation of contact reduction enabled us to respond to urgent requests to actively inform rapidly changing public health policy amidst a pandemic. In early stages, the urgent need was to flatten the curve (Figure 4, panels A, B; Figure 5, panels A, B). Policymakers used initial projections to support decision making on the timing and scope of proposed social distancing policies and to compare potential healthcare need and existing capacity under different scenarios. Once infections began to decrease, there was interest in the degree of increased social contact that could be tolerated as the economy reopened without leading to overwhelmed hospitals (Figure 4, panels C, D; Figure 5, panels C, D). Model estimates were used to evaluate the impact of past policies and to forecast the impact of future proposed interventions, including permutations of layered policies or interventions. Using locally derived estimates enabled policymakers to evaluate potential disease control strategies that were relevant to the current transmission trends in Colorado. For example, as the age distribution shifted within the epidemic in Colorado, estimates with contact rates that varied by age group were produced and used to evaluate policies or interventions targeted to specific age groups, such as return to school and alcohol last call policies.

A key challenge we faced was generating projections of hospital and critical care needs with limited data and rapidly evolving science. Early model estimates were imprecise because data were sparse and poor quality, leading projections to overestimate hospital needs. Estimates and, consequently, projections improved with greater data quantity and quality. Another challenge was the need to generate estimates under extreme time constraints. We designed a preliminary model in a matter of days and adapted it regularly to accommodate new data streams and science. The need for efficiency led to tradeoffs: for example, we did not account for age-specific contact rates (*40*). We present this material not as a finished work but to illustrate how models can be constructed and adapted in real time to inform critical policy questions.

The model findings were provided weekly to decision-makers in Colorado by written reports and briefings. These collaborative interactions provided an opportunity to review findings and define projection scenarios useful for decision-making. The rapidly developed, locally calibrated transmission model provided timely evidence of a moderate decrease in transmission in Colorado after an early transmission control policy began and a substantial decrease in the contact rate after stay-at-home orders that persisted after these policies were partially relaxed. Decreases in transmission mirrored changes in mobility through the end of stay-at-home, at which point mobility ceased to be a clear proxy for transmission. Locally calibrated models have local credibility and can be used to provide time-sensitive, tailored information to policymakers to assist their decision-making.

Appendix Additional information on estimating the impact of statewide policies to reduce spread of severe acute respiratory syndrome coronavirus 2 in real time, Colorado, USA.
