# Supplementary material for: Estimating the Impact of Statewide Policies to Reduce Spread of Severe Acute Respiratory Syndrome Coronavirus 2 in Real Time, Colorado, USA
Source: Emerg Infect Dis. 2021 Sep;27(9):2312–22. doi: 10.3201/eid2709.204167 (PMC8386789; doi:10.3201/eid2709.204167)
Supplement: Appendix — Additional information on estimating the impact of statewide policies to reduce spread of severe acute respiratory syndrome coronavirus 2 in real time, Colorado, USA. [file 20-4167-Techapp-s1.pdf]

Article DOI: https://doi.org/10.3201/eid2709.204167

Estimating the Impact of Statewide Policies to Reduce Spread of Severe Acute Respiratory Syndrome Coronavirus 2 in Real Time, Colorado, USA

Appendix

Case and Hospitalization Data

Two sources of data were used for model fitting. Initially, models were fit to reported cases which were obtained from the Colorado Electronic Disease Reporting System (CEDRS) because hospitalization data were not yet available. CEDRS is updated daily with cases of infection with severe acute respiratory syndrome coronavirus 2 (SARS-CoV-2) cases reported by private laboratories, public laboratories, hospital infection specialists, and state and local public health officials. CEDRS includes information regarding whether SARS-CoV-2 patients were hospitalized.

Starting in May, models were fit to daily SARS-CoV-2 hospital census data. These data were available daily in real-time from EMResource (https://emresource.juvare.com). The EMResource data appear to undercount hospitalizations in March compared with the reported hospitalizations among case-patients in CEDRS (Appendix Figure 1, top panel). On the basis of discussions with health officials and consistent with the literature, we inferred daily hospital census counts by using case data, assuming hospital length was an average of 8 days, starting with report date for hospitalized cases. Because of this discrepancy, we used inferred hospitalizations from the case data through April 7, and switched to EMResource daily hospital census data starting April 8.

Estimating the Start Date of the Epidemic

The first case of COVID19 reported in Colorado was reported on March 5, 2020, and had a reported symptom onset date of February 18, 2020. The next 3 reported cases had a symptom onset date of February 20, 2020. Assuming a 5.1-day incubation period (*1*,*2*), estimates that during the initial phase of the outbreak ≈85% of cases were unreported (*3*), and the outbreak has

a 5.2–6.4 day doubling time (*4*,*5*), we estimated the first cases occurred during January 17–29, 2020.

This estimate was derived by using the number of observed cases on a given day to estimate the number of true cases on the date of observation: $n_t = n_o/u$, where u is the fraction of cases unobserved in early phases of the epidemic. We then used the estimated doubling time (T) to estimate the number of generations required to go from 1 case to n_t such that doubling time, $G = log_2(n_t)$. Because observed cases have an approximate exposure date equal to the onset date (D_o) minus the incubation period (*I*), we estimated the date of first introduction as $D_o - I - T$. We estimated D_o separately for the 1 case that had an onset date of February 18 and the 3 cases that had an onset date of February 20, by using 5.2 and 6.4 as doubling times, yielding estimated date of first infection during January 17–29.

We note that this estimate is consistent with multiple evidence streams indicating introduction of SARS-CoV-2 in the Pacific Northwest in late January to early February (*6*,*7*). Such early introductions are plausible in Colorado, given the high degree of travel into the state during the winter ski season.

Model Details

We provide the model (Appendix Figure 2) and model equations. The model is age-structured and has 3 distinct age groups. The probability that a person becomes infected with SARS-CoV-2 is the same for all age groups. Once a susceptible person is infected, he or she moves to the exposed compartment in which they are not yet infectious. After spending time in the exposed compartment, a person can either be infectious and symptomatic or infectious and asymptomatic. We provide probabilities that an exposed person has symptoms and probabilities that a symptomatic person requires hospitalization that is age dependent (Appendix Table 1). Symptomatic persons are assumed to be infectious and presymptomatic for 1 of the 8 days they are infectious. On the basis of evidence suggesting that asymptomatic persons are less infectious than symptomatic persons (*11*), a parameter is included describing the ratio of infectiousness for symptomatic versus asymptomatic persons. We provide this parameter and other key model parameters (Appendix Table 2). It is assumed no further transmission occurs once the patient enters the hospital.

Model Equations

The susceptible-exposed-infected-recovered model describes the movement of the population of Colorado (N) through the following model compartments: susceptible (S), exposed (E), infectious and symptomatic (I), infectious and asymptomatic (A) recovered (R), hospitalized (Ih), needing care in an intensive care unit (ICU) (Ic), recovered from hospitalization or ICU (Rh and Rc), and deaths (D). The model is age stratified, such that there are compartments for each of the 3 age strata (i) (<30, 30–59, $\geq$60 years). The subscripts indicate the age strata for each variable. For example, A_2 is the number of asymptomatically infected persons in the second age class (30–59 years). Similarly, $\sum_{j=1}^{3} I_j$ is the sum of infected persons across the 3 age classes. Mask wearing ($mask_t$), self-isolation of infectious persons (siI_t) and social distancing (SD_t) metrics are assumed to change over time, as indicated by the subscript.

$$\frac{dS_i}{dt} = -\frac{\beta}{N} * \lambda * (1 - mask_t * 0.03) * (1 - siI_t) * (1 - SD_t) * S_i * \sum_{j=1}^{3} I_j - \frac{\beta}{N} * (1 - mask_t * 0.27) * (1 - SD_t) * S_i * \sum_{j=1}^{3} A_j$$

$$\frac{dE_i}{dt} = \frac{\beta}{N} * \lambda * (1 - mask_t * 0.03) * (1 - siI_t) * (1 - SD_t) * S_i * \sum_{j=1}^{3} I_j + \frac{\beta}{N} * (1 - mask_t * 0.27) * (1 - SD_t) * S_i * \sum_{j=1}^{3} A_j - \frac{1}{\alpha} * E_i$$

$$\frac{dI_i}{dt} = \frac{pS_i}{\alpha} * E_i - \frac{I_i}{\gamma}$$

$$\frac{dIh_i}{dt} = \frac{I_i}{\gamma} * hosp_i - \frac{Ih_i}{losh}$$

$$\frac{dIc_i}{dt} = \frac{I_i}{\gamma} * cc_i - \frac{Ic_i}{losc}$$

$$\frac{dA_i}{dt} = \frac{(1 - pS_i)}{\alpha} * E_i - \frac{A_i}{\gamma}$$

$$\frac{dR_i}{dt} = \frac{A_i + I_i(1 - hosp_i - cc_i)}{\gamma}$$

$$\frac{dRh_i}{dt} = \frac{Ih_i}{losh}$$

$$\frac{dRc_i}{dt} = \frac{(1-d) * Ic_i}{losc}$$

$$\frac{dD_i}{dt} = \frac{d * Ic_i}{losc}$$

The effective reproductive number (R_e) was estimated by using the following equation:

$$R_e = \frac{\frac{\sum_{j=1}^{3} E_{j_t}}{\alpha}}{\frac{\sum_{j=1}^{3} I_{j_{t-\alpha-1}}}{\gamma}}$$

Model Identifiability

Analysis was performed using the computation in Omlin et al. (*13*). In this study, we have fit a social distancing parameter that switches at 4 different timepoints in the course of the hospitalization time series. A natural question is whether or not these 4 parameters are identifiable given the data. In Omlin et al. (*13*), the authors describe a colinearity index (gamma), which measures how the change in 1 parameter can be compensated by the fraction 1 − 1/gamma change in other parameters. The gamma threshold for identifiability is conventionally set in the range of 10 to 15 (for gamma = 10, 1 – 1/gamma = 90%). We considered all possible combinations of 1, 2, 3, and 4 social distancing parameters and show the threshold (Appendix Figure 3). There is no issue with identifiability when trying to estimate ≤3 parameters. The collinearity of identifying all 4 parameters is ≈10, which is right on the edge of acceptability. In practice, we have not found this to be a problem because when we consider more recent hospitalization data, the previously estimated social distancing estimates do not change appreciably.

Data and Code

Model code, parameters, and data used to inform the fitting process can be found at https://github.com/agb85/CFpaper

References

1. Lauer SA, Grantz KH, Bi Q, Jones FK, Zheng Q, Meredith HR, et al. The incubation period of coronavirus disease 2019 (COVID-19) from publicly reported confirmed cases: estimation and application. Ann Intern Med. 2020;172:577–82. PubMed https://doi.org/10.7326/M20-0504

2. Li Q, Guan X, Wu P, Wang X, Zhou L, Tong Y, et al. Early transmission dynamics in Wuhan, China, of novel coronavirus-infected pneumonia. N Engl J Med. 2020;382:1199–207. PubMed https://doi.org/10.1056/NEJMoa2001316

3. Li R, Pei S, Chen B, Song Y, Zhang T, Yang W, et al. Substantial undocumented infection facilitates the rapid dissemination of novel coronavirus (SARS-CoV-2). Science. 2020;368:489–93. PubMed https://doi.org/10.1126/science.abb3221

4. Wu JT, Leung K, Bushman M, Kishore N, Niehus R, de Salazar PM, et al. Estimating clinical severity of COVID-19 from the transmission dynamics in Wuhan, China. Nat Med. 2020;26:506–10. PubMed https://doi.org/10.1038/s41591-020-0822-7

5. Wu JT, Leung K, Leung GM. Nowcasting and forecasting the potential domestic and international spread of the 2019-nCoV outbreak originating in Wuhan, China: a modelling study. Lancet. 2020;395:689–97. PubMed https://doi.org/10.1016/S0140-6736(20)30260-9

6. Centers for Disease Control and Prevention. First travel-related case of 2019 novel coronavirus detected in United States, January 21, 2020 [cited 2021 Jun 3]. https://www.cdc.gov/media/releases/2020/p0121-novel-coronavirus-travel-case.html

7. Bedford T, Greninger AL, Roychoudhury P, Starita LM, Famulare M, Huang ML, et al.; Seattle Flu Study Investigators. Cryptic transmission of SARS-CoV-2 in Washington state. Science. 2020;370:571–5. PubMed https://doi.org/10.1126/science.abc0523

8. Davies NG, Klepac P, Liu Y, Prem K, Jit M, Eggo RM; CMMID COVID-19 working group. Age-dependent effects in the transmission and control of COVID-19 epidemics. Nat Med. 2020;26:1205–11. PubMed https://doi.org/10.1038/s41591-020-0962-9

9. Ferguson N, Laydon D, Nedjati-Gilani G, Imai N, Ainslie K, Baguelin M, et al. Report 9: impact of non-pharmaceutical interventions (NPIs) to reduce COVID-19 mortality and healthcare demand, 2020 [cited 2021 Jun 3]. https://www.imperial.ac.uk/media/imperial-college/medicine/sph/ide/gida-fellowships/Imperial-College-COVID19-NPI-modelling-16-03-2020.pdf

10. Bi Q, Wu Y, Mei S, Ye C, Zou X, Zhang Z, et al. Epidemiology and transmission of COVID-19 in 391 cases and 1286 of their close contacts in Shenzhen, China: a retrospective cohort study. Lancet Infect Dis. 2020;20:911–9. PubMed https://doi.org/10.1016/S1473-3099(20)30287-5

11. He X, Lau EH, Wu P, Deng X, Wang J, Hao X, et al. Temporal dynamics in viral shedding and transmissibility of COVID-19. Nat Med. 2020;26:672–5. PubMed https://doi.org/10.1038/s41591-020-0869-5

12. Cheng HY, Jian SW, Liu DP, Ng TC, Huang WT, Lin HH; Taiwan COVID-19 Outbreak Investigation Team. Contact tracing assessment of COVID-19 transmission dynamics in Taiwan and risk at different exposure periods before and after symptom onset. JAMA Intern Med. 2020;180:1156–63. PubMed https://doi.org/10.1001/jamainternmed.2020.2020

13. Omlin M, Brun R, Reichert P. Biogeochemical model of Lake Zürich: sensitivity, identifiability and uncertainty analysis. Ecol Modell. 2001;141:105–23. https://doi.org/10.1016/S0304-3800(01)00257-5

Appendix Table 1. Estimated probability that an exposed (and latently infected) individual will be symptomatic, and a symptomatic individual will need hospital care*

Age group, years	Probability of symptoms, given infection	Probability of non-ICU hospitalization given symptomatic COVID-19	Probability of needing ICU care given symptomatic COVID-19
Colorado specific†			
<30	NA	0.00419	0.004
30–59	NA	0.056	0.019
≥60	NA	0.089	0.035
From the literature‡			
<30	0.18	0.006	0.0003
30–59	0.47	0.059	0.0045
≥60	0.79	0.207	0.0808

*The probability that an exposed person becomes symptomatic is based on the literature and adjusted to the age structure of the Colorado population. The probability a symptomatic person requires hospital care is based on Colorado case and hospitalization data and compared with estimates obtained from the literature. COVID-19, coronavirus disease; ICU, intensive care unit.
†The Colorado estimates were based on 2-part optimization by using Colorado hospitalization data and our age-structured susceptible-exposed-infected-recovered model. Colorado-specific estimates were only possible to derive with sufficient data starting in May and were incorporated into the model from then onwards (fits 3 and 4) in place of the literature-derived estimates.
‡Age-dependent probabilities that an infected person is symptomatic are based on estimates of Davies et al. (*8*). Age-dependent probabilities of hospitalization and ICU need are based on estimates summarized by (*9*). Because the age compartments of published estimates do not precisely align with our age categories, these estimates are a weighted sum of the age-specific probability of symptoms and the proportion of the population of Colorado within each age-compartment. Colorado demographic data of estimated population distribution in 2020 were based on US census projections. Literature-derived estimates of age-specific hospitalization and ICU need were used for fit 1 and fit 2. Literature-derived age-specific symptomatic fraction was used for all fitting.

Appendix Table 2. Parameters in model and value estimates and sources*

Symbol	Description	Estimate	Source
β	Transmission rate: equivalent to combination of transmission probability and contact rate	Estimated from model	Model
λ	Ratio of infectiousness for symptomatic vs. asymptomatic persons. Evidence suggests that asymptomatic persons are less infectious than symptomatic persons (*10*). However, the comparative infectiousness is unknown.	Estimated from model	Model
$mask$	Proportion of population wearing masks	Estimated from model	Model
siI	Reduction in infectious contacts due to symptomatic persons who self-isolate after March 5, 2020	Estimated from model	Model
SD	Reduction in infectious contacts due to social distancing and other transmission reducing measures	Estimated from model	Model
α	Incubation period	5.1 d	(*1,10*)
pS_i	Age-specific probability of development of symptoms if infected	0.18 (age 0–29 years), 0.47 (age 30–59 years), 0.79 (age ≥60 years)	(*8*)
γ	Recovery rate or duration of infectiousness	8 d	(*11,12*)
pID	Probability symptomatic cases are identified by state surveillance	Estimated from model	Model
$hosp_i$	Age-specific probability of non-ICU hospitalization, given symptomatic infection	Appendix Table 1	NA
losh	Non-ICU hospitalization length of stay	8 d	(*9*)
cc_i	Age-specific probability of ICU-hospitalization, given symptomatic infection	Appendix Table 1	NA
losc	ICU hospitalization length of stay	10 d	(*9*)
D	Probability of death, given ICU hospitalization	0.5	(*9*)
N	Total population	5,840,795	CDPHE

*CDPHE, Colorado Department of Public Health and Environment; ICU, intensive care unit. NA, not applicable

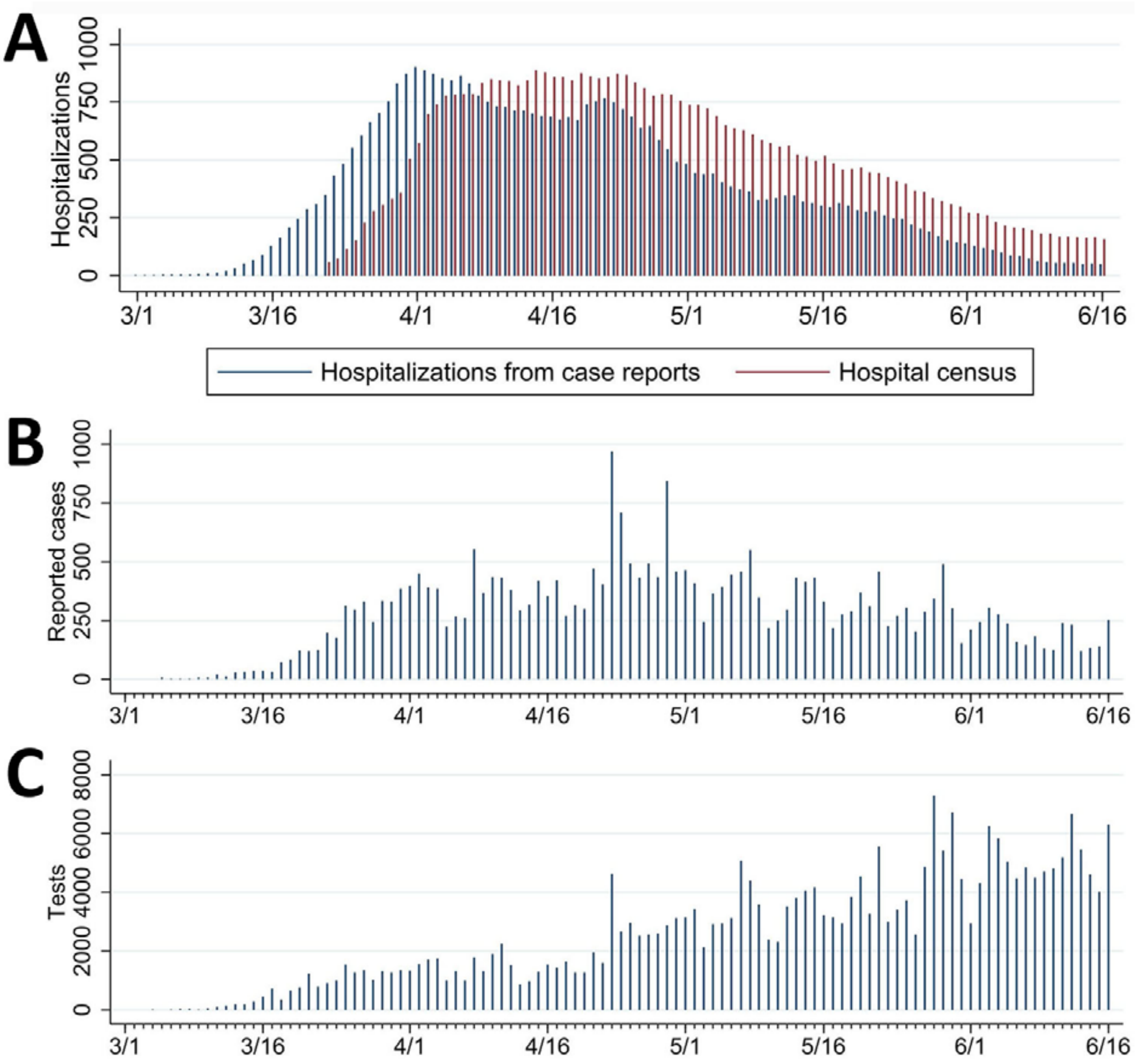

Appendix Figure 1. Top: daily number of hospitalized coronavirus disease patients based on Colorado Department of Public Health and Environment (blue lines) and EMResource (https://emresource.juvare.com) hospital census data (red lines). Middle: Severe acute respiratory syndrome coronavirus 2 cases reported to the state health department (Colorado Department of Public Health and Environment) by report date. Bottom: reported number of severe acute respiratory syndrome coronavirus 2 tests performed per day in Colorado.

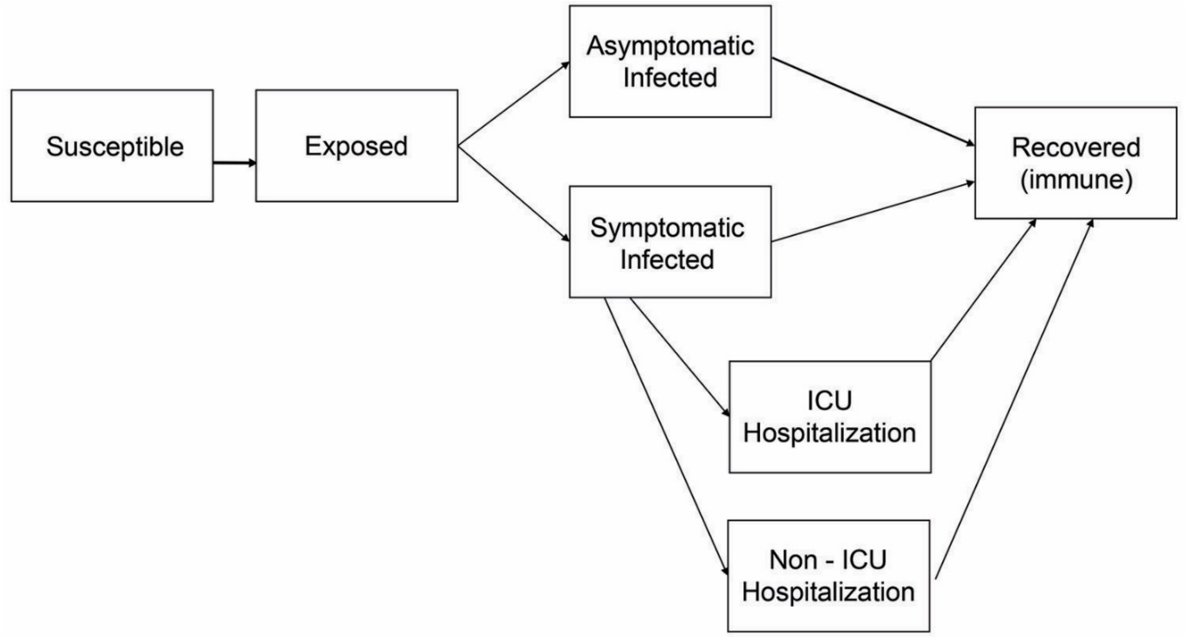

Appendix Figure 2. Structure of the age-structured susceptible-exposed-infected-recovered model. Model includes separate compartments for the 3 age groups: 0–29 years, 30–59 years, and $\geq$60 years. ICU, intensive care unit.

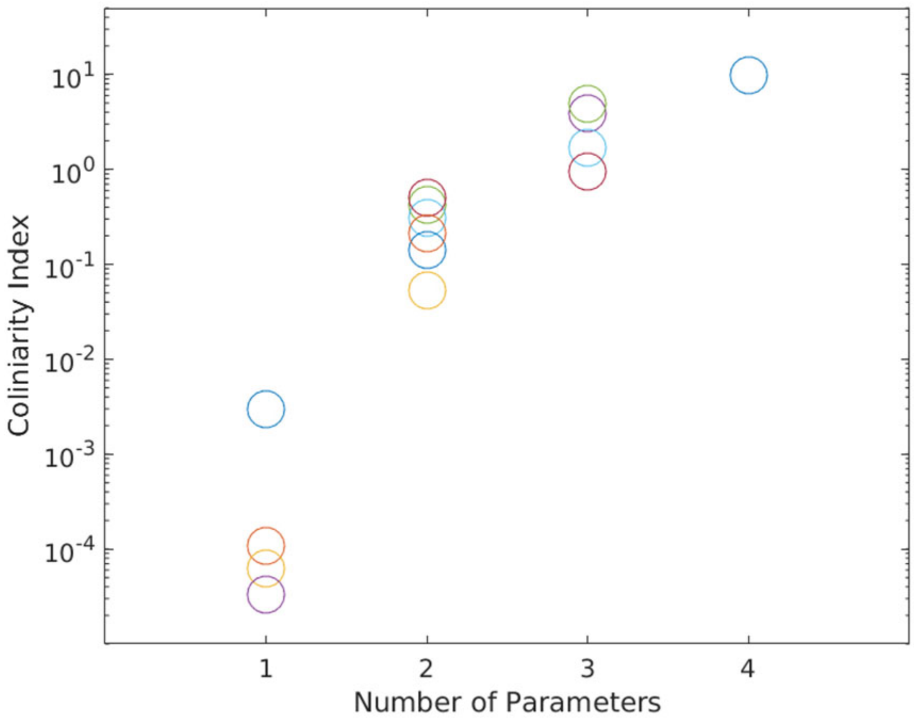

Appendix Figure 3. Collinearity plot from identifiability analysis. In this study, we have fit a social distancing parameter that switches at 4 different timepoints in the course of the hospitalization time series.
